# Effects of ultraviolet radiation on the activity, survival, and growth of the critically endangered northern corroboree frog

**DOI:** 10.1242/bio.061827

**Published:** 2025-05-13

**Authors:** Michael S. McFadden, Aimee J. Silla, Shannon R. Kelleher, Phillip G. Byrne

**Affiliations:** ^1^ Herpetofauna Department, Taronga Conservation Society Australia, PO Box 20, Mosman, NSW 2088, Australia; ^2^ Environmental Futures, School of Science, The University of Wollongong, Wollongong NSW 2522, Australia

**Keywords:** Ultraviolet radiation, UVR, Captive breeding, Amphibian, Fitness, Behaviour, Conservation, Threatened species

## Abstract

Ultraviolet radiation (UVR) is known to have lethal and sublethal impacts on amphibians, yet effects on anuran growth, development and behaviour remain understudied. As the global amphibian extinction crisis worsens, and more species enter conservation breeding programs (CBPs), there is a growing need to understand interrelationships between UVR exposure, growth, and UVR avoidance behaviour. Here, we investigate the effect of ecologically appropriate UVR levels on post-metamorphic growth and activity in the critically endangered northern corroboree frog (*Pseudophryne pengilleyi*). UVR was provided at one of two levels (lower UVI, ∼0.2 and higher UVI, ∼0.75), for a period of 16 weeks, spanning the first growth phase before hibernation. Daily activity was significantly lower in frogs reared under the higher UVR treatment (mean±s.e.m=26.339%±0.757) compared to those receiving the lower UVR treatment (mean±s.e.m=35.660%±0.837). Despite these differences, there was no significant difference between treatment groups in survival or growth. These findings indicate that northern corroboree frogs may have the capacity to adjust their behaviour in response to UVR and suggest that behavioural plasticity might mitigate potential negative impacts of UVR exposure. We discuss the value of this knowledge for amphibian CBPs.

## Teaser

**Summary:** Ecologically appropriate levels of UVR influence activity, but not survival or growth, in the critically endangered northern corroboree frog. This knowledge is expected to assist with conservation breeding.

## INTRODUCTION

Ultraviolet radiation (UVR) triggers various photochemical reactions that are essential for life, including vitamin D synthesis, photo enzymatic DNA repair, and vision (Malloy et al., 1997; Alton and Franklin, 2017). Yet, for many organisms, excess exposure to UVR can have harmful effects that reduce lifetime fitness (Alton and Franklin, 2017; Fu et al., 2022). At a molecular level, UVR damage to lipids, proteins and DNA can severely compromise cellular function, with lethal consequences (Alton and Franklin, 2017). However, lifetime fitness might also be negatively impacted through various sublethal effects, including changes to behaviour, locomotion, cardiorespiratory function, hormone-stress response, oxidative stress response, immune function and disease susceptibility (Cramp and Franklin, 2018; Downie et al., 2023). Of growing interest is the impact of UVR exposure on organismal growth and development (Croteau et al., 2008)**.** In principle, while growth might be directly slowed by delays to DNA transcription and replication followed by programmed cell death (apoptosis) (Alton and Franklin, 2017), we can also expect that growth and development will be altered by indirect effects.

In response to high UVR exposure many organisms employ a range of physiological defence mechanisms to minimise harmful impacts. Common defences include the production of protective UVR screening compounds (e.g. melanin), the activation of heat shock proteins, DNA repair mechanisms, and increased protein and lipid synthesis (Alton and Franklin, 2017; Blaustein and Belden, 2003). Because these responses all carry high energetic costs that cause energy trade-offs (Alton et al., 2012; Cramp et al., 2022; Fischer et al., 2006; García-Huidobro et al., 2017; Kazerouni et al., 2016; Malloy et al., 1997; Snell et al., 2009; Vargas et al., 2018), they are expected to significantly slow organismal growth and development (Croteau et al., 2008). Importantly, the extent of these costs can potentially be mitigated through behavioural responses. In various taxa, individuals stand to reduce UVR exposure and ensure optimal growth and development through behavioural avoidance of high UVR microhabitats (Blaustein and Belden, 2003; Pulgar et al., 2015; Sigg et al., 2007; Tang et al., 2011; Vargas et al., 2018). This adaptation appears to be particularly important for ectotherms that must balance gaining heat from solar radiation against avoiding levels of UVR exposure that might be harmful (Croteau et al., 2008).

Among amphibians, research into the impacts of UVR on individual survival and performance has increased exponentially in the past three decades, largely through a push to explain the rapid decline of amphibians globally (Croteau et al., 2008; Alton and Franklin, 2017; Lundsgaard et al., 2020; Ceccato et al., 2016; Cramp et al., 2022). Major amphibian declines were first detected in the 1980s, and 40.7% of the worlds' amphibians are now threatened (Luedtke et al., 2023). While the spread of infectious diseases and habitat loss are believed to be key drivers of decline (Luedtke et al., 2023), climate change and negative impacts of UVR exposure also appear to be contributing, with strong evidence for UVR-induced mortality across all amphibian life stages (Belden et al., 2000; Belden and Blaustein, 2002b; Blaustein and Belden, 2003; Blaustein and Kiesecker, 2002; Fite et al., 1998; Hays et al., 1996; Hofer and Mokri, 2000; Lundsgaard et al., 2020). In addition, various sublethal impacts of UVR exposure have been reported, including malformations (Ankley et al., 2000; Fite et al., 1998), increased predation risk (Alton et al., 2011), developmental abnormalities (Worrest and Kimeldorf, 1976), decreased immune function (Ceccato et al., 2016; Cramp et al., 2022) and delayed growth (Belden et al., 2000; Belden and Blaustein, 2002a,b; Pahkala et al., 2001a).

As amphibian declines worsen, the establishment of conservation breeding programs (CBPs) to safeguard threatened species has become an increasingly valuable *ex situ* conservation action (Bradfield et al., 2023). This global initiative has spurred new empirical research focused on developing optimal husbandry practices, including UVR conditions for rearing amphibians in captivity (Michaels et al., 2015; Tapley et al., 2015; Verschooren et al., 2011; Antwis et al., 2014; Newton-Youens et al., 2022; Whatley et al., 2020). Knowledge in this area is proving to be crucial to the success of amphibian CBPs because attempts to raise many species have been marred by UVR-related issues (Antwis and Browne, 2009). One of the most widespread is the onset of metabolic bone disease, a weakening of the bones resulting from inappropriate UV-B light provision during larval and post metamorphic development combined with diets low in calcium and vitamin D_3_ (Antwis and Browne, 2009). Despite an increasing need to identify optimal UVR conditions for rearing captive amphibians, little is known about the sublethal effects of ecologically appropriate UVR exposure regimes, with a deficit of information on behavioural responses to varying UVR levels across species.

Observations of amphibians in the wild have reported that UVR influences micro-habitat selection (Kats et al., 2012), and manipulative laboratory experiments have indicated that certain species (particularly those that are diurnally active) show a behavioural preference for lower levels of UV-B (DeMarchi et al., 2018; Han et al., 2007; Kats et al., 2012; Michaels and Preziosi, 2013; Van de Mortel and Buttemer, 1998). Based on this work, designing CBPs that enable behavioural regulation of UVR exposure may maximise the benefits of UVR exposure, while minimising lethal and sublethal effects (Alton and Franklin, 2017; Cramp et al., 2022). However, given that amphibians are adapted to a diversity of environments (ranging from low UVR tropical rainforests to high UVR alpine areas) and display species-specific differences in both their physiological defence capacity (Hird et al., 2022) and activity patterns (ranging from nocturnal to diurnal), it may be difficult to generalise about the effects of UVR exposure across species. As such, for any threatened species where CBPs have been established, there will be value in investigating relationships between UVR exposure, activity behaviour, growth, and development.

**Fig. 1.**
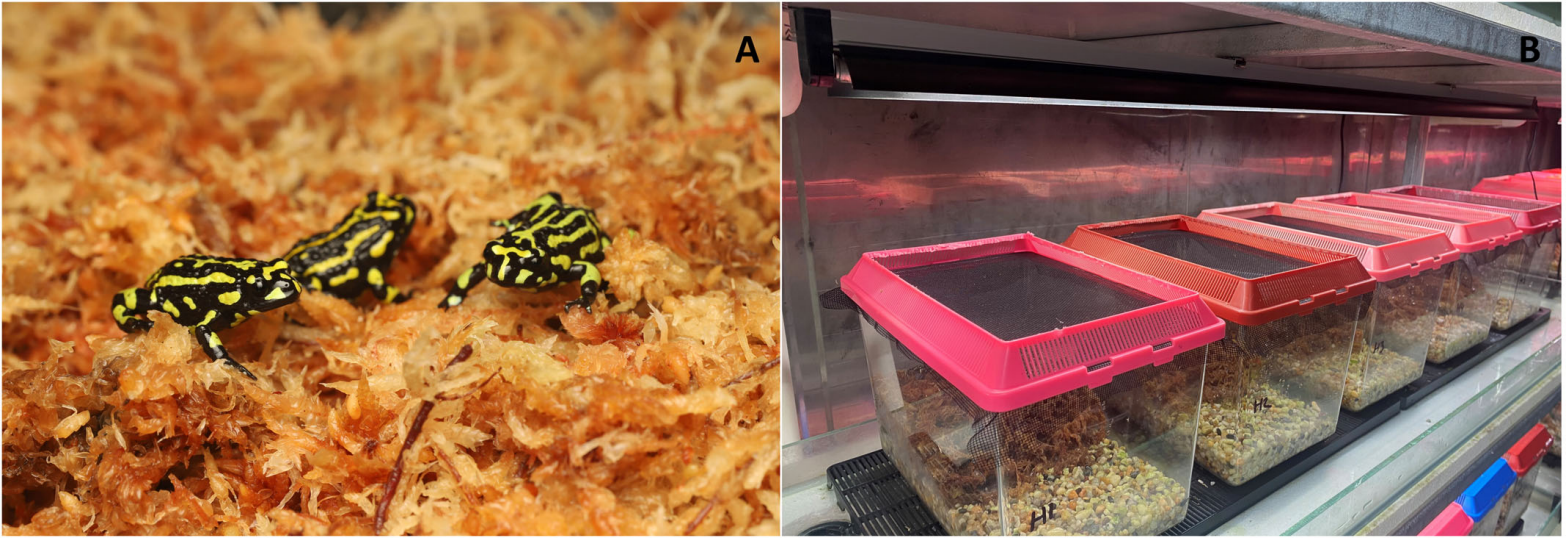
**(A) Northern corroboree frog (*P. pengilleyi*) metamorphs on sphagnum moss substrate, housed in groups of five individuals per enclosure for the duration of the 16-week study.** The two experimental treatments (lower UVR or higher UVR) each had ten containers, equating to 50 individuals per treatment group. (B) Experimental enclosures were secured with a ventilated flyscreen mesh lid that allowed for UV-B light penetration. Photographs courtesy of Michael McFadden.

In Australia, amphibian declines have been substantial, driven primarily by the amphibian chytrid fungus, *Batrachochytrium dendrobatidis* (*Bd*), bush fires, drought, invasive species, and habitat loss. In recent decades, a total of five species have gone extinct, and 17% of all remaining species (40/238) are threatened with extinction (Gillespie et al., 2020). In response to these declines, CBPs have been established for 14 species (McFadden et al., 2018). This includes the iconic and critically endangered corroboree frogs, comprised of the southern corroboree frog (*Pseudophryne corroboree*) and the northern corroboree frog (*Pseudophryne pengilleyi*). While the CBPs for these species have successfully generated hundreds of frogs for reintroduction and maintained effective captive assurance populations (McFadden et al., 2018), knowledge of impacts of UVR exposure on performance and fitness is lacking. This knowledge gap highlights a need for experimental research aimed at investigating corroboree frog responses to varying UVR levels. Ultimately, experimental research will help to identify captive-rearing conditions that maximise the benefits of UVR exposure, while minimising lethal and sublethal effects.

The aim of the present study was to conduct a manipulative laboratory experiment with captive northern corroboree frogs to investigate the influence of different levels of UVR on sub adult activity, survival, and growth. Corroboree frogs inhabit high UVR sub-alpine habitats, and while they are primarily nocturnal in the wild, diurnal activity is observed on overcast days when UVR is low (D. Hunter, personal communication). Based on this apparent avoidance of high UVR conditions, we predicted that captive northern corroboree frogs would be less active under conditions of higher UVR, and that this behaviour would mitigate any potentially negative impacts of UVR exposure on growth and survival.

## RESULTS

### Survival

By the end of the experimental period (week 16), 100% of experimental animals had survived, with no individuals displaying any observable abnormalities.

### Activity

**Fig. 2.**
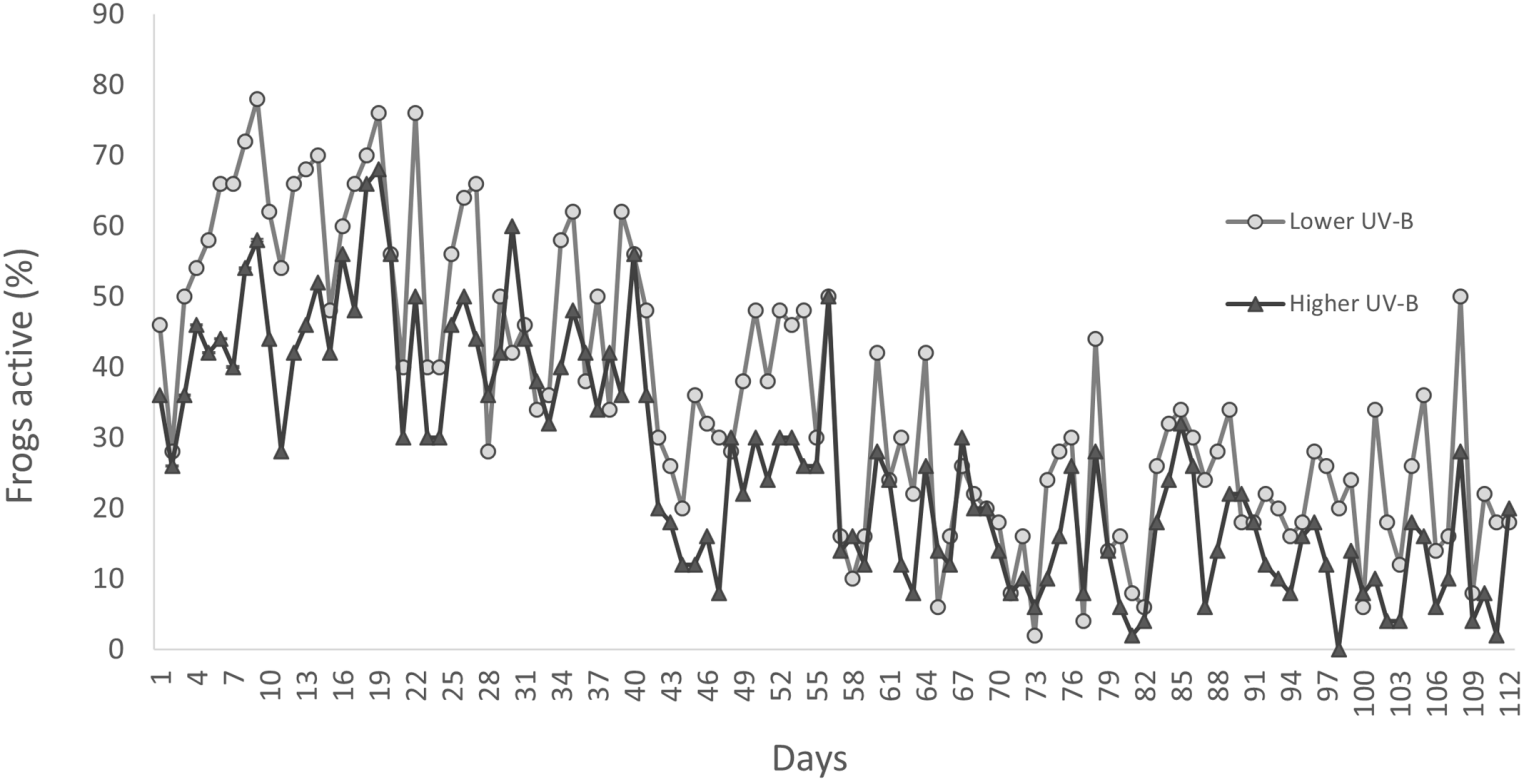
**Effect of UVR treatment (lower UVR and higher UVR) on the mean percentage of frogs active over 112 consecutive days in the northern corroboree frog *P. pengilleyi* (*n*=50 individuals per treatment).** The reduction in activity over time was significant for both treatment groups (LME: *F_1,2219_*=772.38, *P*<0.0001), as was the difference in activity level between treatment groups (LME: *F_1,18_*=6.95, *P*=0.017).

Over 112 consecutive days, frogs in both UVR treatment groups displayed a decline in activity (Fig. 2). On days 1-14, the mean percentage (± s.e.m.) of frogs active on the surface was 42.43%±1.9 (range=0%-100%) for the higher UVR treatment, and 59.86%±2.02 (range=0%-100%) for the lower UVR treatment. On days 99-112, the mean percentage of active frogs was 10.86%±1.37 (range=0% and 80%) for the higher UVR treatment and 21.57%±1.81 (range=0%-100%) for the lower UVR treatment. The reduction in activity over time was significant for both treatment groups (LME: *F_1,2219_*=772.38, *P*<0.0001). Over the entire experimental period, activity levels (proportion of frogs active on the surface) were lower in frogs from the higher UVR treatment group (mean±s.e.m.=26.339%±0.757) compared to frogs from the lower UVR treatment group (mean±s.e.m.=35.660%±0.837) (Fig. 2). Overall, the difference in activity level between treatment groups was statistically significant (LME: *F_1,18_*=6.95, *P*=0.017).

### Body mass

**Fig. 3.**
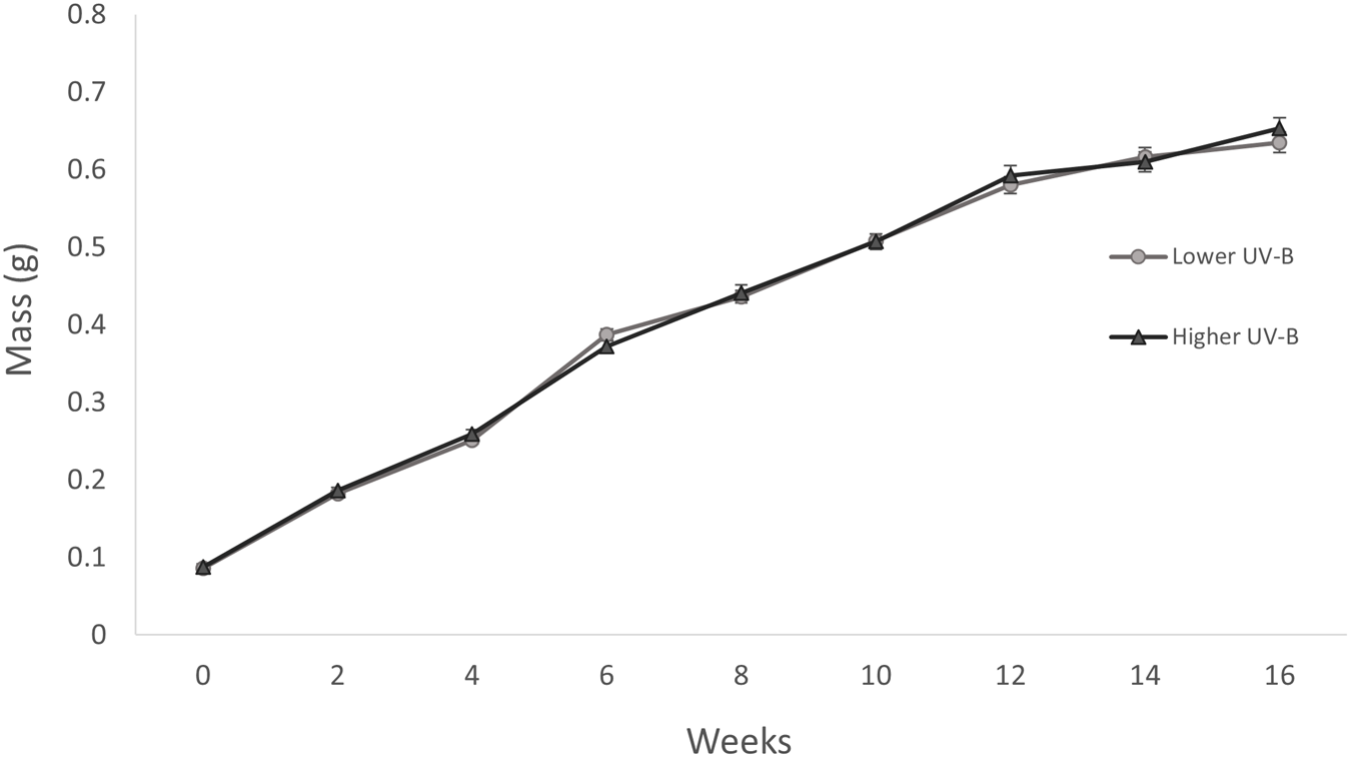
**Effect of UVR treatment (lower UVR and higher UVR) on metamorph body mass over 16 experimental weeks in the northern corroboree frog *P. pengilleyi* (*n*=50 individuals per treatment).** Data presented are untransformed mass (g), mean±s.e.m. For both treatment groups, there was a significant change in body mass across experimental weeks (LME: *F_7,693_*=0.578, *P*<0.0010) but there was no significant difference between the UVR treatment groups (LME: *F_1,98_*=0.492, *P*=0.484).

On experimental day 1, there was no difference in mean body mass between the two UVR treatment groups (one-way ANOVA: *F_1,98_*=0.2153, *P*=0.643, mean±s.e.m. lower UVR=0.086±0.003, higher UVR=0.088±0.003). Over the 16-week study period body mass increased consistently, with frogs displaying more than an 8-fold increase in weight between the start and the end of the study (Fig. 3). While the change on body mass over across experimental weeks was significant (LME: *F_7,693_*=0.578, *P*<0.0010), there was no significant difference between the UVR treatment groups (LME: *F_1,98_*=0.492, *P*=0.484).

### Body length (SVL)

**Fig. 4.**
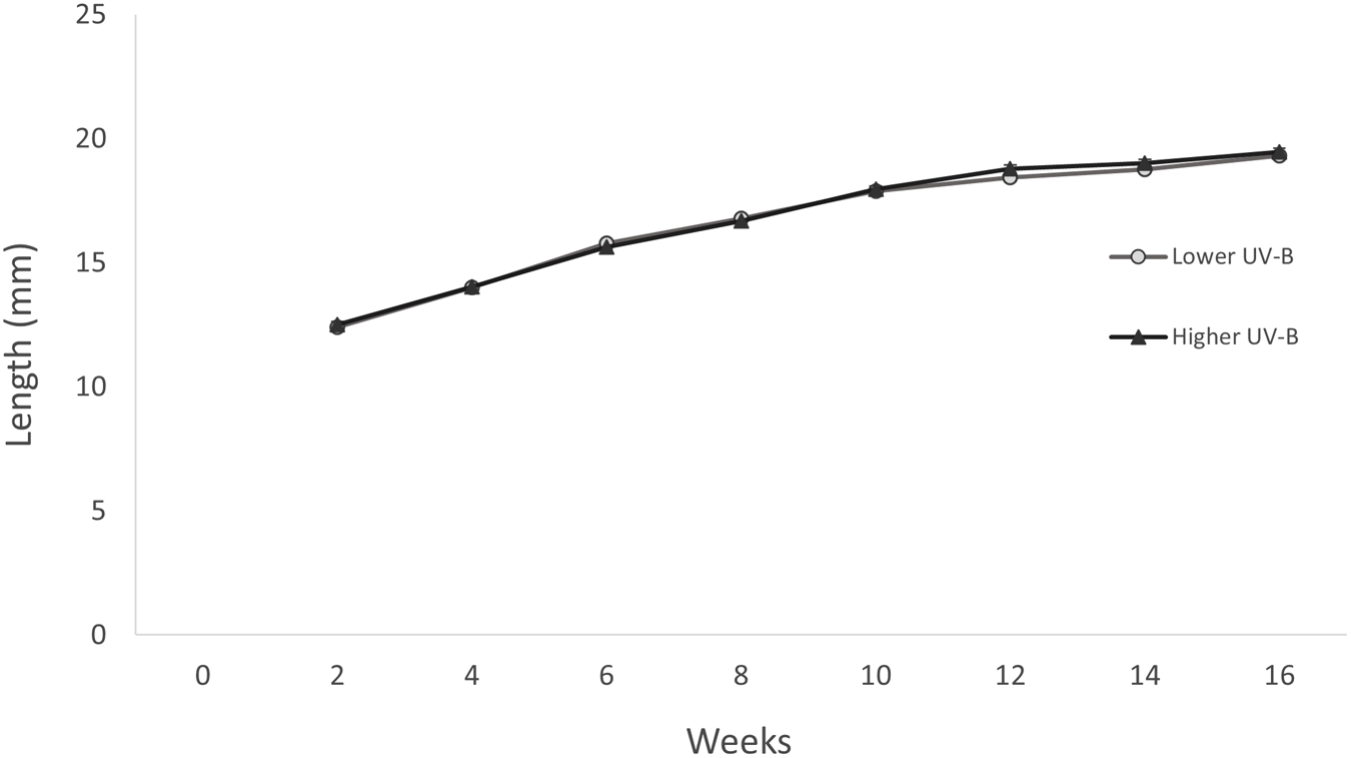
**Effect of UVR treatment (lower UVR and higher UVR) on metamorph snout-vent length over 16 experimental weeks in the northern corroboree frog *P. pengilleyi* (*n*=50 individuals per treatment).** Data presented are untransformed length (mm), mean±s.e.m. For both treatment groups, there was a significant change in body length across experimental weeks (LME: *F_7,693_*=1257.79, *P*<0.0010) but there was no significant difference between the UVR treatment groups (LME: *F_1,98_*=0.964, *P*=0.329).

On experimental day 1, there was no difference in mean body length between the two UVR treatment groups (one-way ANOVA: *F_1,98_*=0.155, *P*=0.695, mean±s.e.m. lower UVR=10.036±0.081, higher UVR=10.088±0.100). Over the 16-week study period body length (mm) increased consistently, with frogs almost doubling in snout-vent length (SVL) between the start and the end of the study (Fig. 4). While the change on body length over across experimental weeks was significant (LME: *F_7,693_*=1257.79, *P*<0.0010), there was no significant difference between the UVR treatments (LME: *F_1,98_*=0.964, *P*=0.329).

## DISCUSSION

As the global amphibian extinction crisis worsens, and more threatened species enter CBPs, the influence of UVR on amphibian survival, growth and development is increasingly being investigated. Surprisingly, however, few studies have attempted to identify optimal levels of UVR provision or considered whether lethal and sublethal impacts of UVR exposure might be mediated through behavioural responses (Bancroft et al., 2008; Kats et al., 2012, 2000; Michaels and Preziosi, 2013; Pahkala et al., 2003). The aim of the present study was to conduct a manipulative laboratory experiment with captive northern corroboree frogs (*P. pengilleyi*) to investigate the influence of ecologically appropriate levels of UVR on post-metamorphic activity, survival, and growth. We predicted that frogs would be less active in the higher UVR treatment (which sits at the top end of UVR conditions likely to be experienced by *P*. *pengilleyi* in the wild), and that changes to behaviour would mitigate any potentially negative impacts of UVR exposure on growth and survival. Our results show that there was a statistically significant effect of UVR treatment on activity, whereby the proportion of frogs observed on the substrate surface was significantly lower in the higher UVR treatment (∼0.75 UVI level) than the lower UVR treatment (∼0.2 UVI level). In addition, our results show that there was no effect of UVR treatment on frog survival or growth over the experimental period.

### UVR and activity

Our finding that frog activity was significantly lower under the higher UVR conditions supported our hypothesis that northern corroboree frogs have evolved the capacity to behaviourally avoid higher and potentially harmful UV conditions. However, because we only had two treatment levels, and did not conduct UVR preference tests, we cannot rule out the possibility that frogs in the lower UVR treatment were displaying elevated activity. Specifically, it could be argued that frogs under the lower UVR conditions were receiving suboptimal amounts of UVR and were stimulated to spend more time on the substrate surface to maximise UV exposure. At present, we have no data on ambient UV-A and UV-B levels in corroboree frog natural habitats, or variation in UVR levels within and between microhabitats used for shelter, foraging and breeding. However, it must be emphasised that the UVR level provided in the lower-UVR treatment was within the natural range expected for an ectothermic species inhabiting a low UVR environment. Although relationships between UVR levels in natural habitats and activity schedules are yet to be clearly established for amphibians, relevant information is available for reptiles. Ferguson et al. (2010) studied 15 lizard and snake species in the field and identified four discrete UVR exposure groups. These being: 1) species that are crepuscular or shade dwelling (UVI range=0-0.7), 2) species residing in partial sun and occasionally basking (UVI range=0.7-1.0), 3) species seeking partial sun and basking (UVI range=1.0-2.6), and 4) species seeking mid-day sun and basking (UVI range=2.6-3.5). In the wild, corroboree frogs are primarily nocturnal, yet activity still occurs at dawn and dusk, and during the day when conditions are overcast (D. Hunter, personal communication). Therefore, we can assume that frogs in the higher UVR treatment were receiving radiation levels towards the top end of the range that the species is adapted to. Bearing in mind that even ambient levels of UVR are known to have lethal and sublethal consequences for amphibians (Belden and Blaustein, 2002b; Blaustein et al., 2005; Häkkinen et al., 2001), the most likely explanation for our results is that frogs in the higher UVR treatment were reducing their activity to avoid excessive UVR exposure (Han et al., 2007). Saying this, we note that incident light levels were higher in the higher UVR treatment than the lower UVR treatment. This raises the possibility that frogs were assessing and avoiding high light levels rather than UVR, potentially to mitigate predation risk and/or manage thermoregulatory requirements. This point aside, our supposition that northern corroboree frogs are sensitive to UVR is further supported by knowledge that captive corroboree frogs will incur skin damage at UVI levels associated with exposure to the partial shade or open sun (>1.4 UVI) (M. McFadden, unpublished data).

Regardless of whether frogs were avoiding or seeking UVR, our behavioural data indicate that northern corroboree frogs have the capacity to assess and respond to different levels of environmental UVR (or incident light levels correlated with higher UVR). This is an important finding because it adds to an emerging body of evidence that anuran amphibians can behaviourally modulate exposure to potentially harmful ultraviolet radiation. A combination of field observations, field experiments, and behavioural-choice tests have revealed a preference for lower UVR environments in various frog species. These include the neotropical poison-dart frog (*Dendrobates pumilio*) (DeMarchi et al., 2018; Han et al., 2007; Kats et al., 2012), the neotropical green poison-dart frog (*D. auratus*) (Han et al., 2007), Bransford's litter frog *Craugastor bransfordii* (DeMarchi et al., 2018), the European pool frog (*Pelophylax lessonae*) (Michaels and Preziosi, 2013), larvae of the Australian green and golden bell frog (*Litoria aurea*) (Van de Mortel and Buttemer, 1998), and larvae of the emerald treefrog (*Litoria peronii*) (Van de Mortel and Buttemer, 1998). Interestingly, these findings all come from studies in diurnally active species that experience consistently high UVR levels, implying that there may be strong selection for behavioural regulation of UVR exposure in such groups. The fact that our findings suggest similar behaviour in a cryptic primarily nocturnal frog species indicates that the capacity for behavioural regulation of UVR exposure may be more widespread among anurans than currently realised. Northern corroboree frogs have evolved in high elevation sub-alpine environments, and such locations are known to receive very high levels of UV radiation. As a rule, UV-B levels rise by ∼10% with every 1000 m in altitude (Schmucki and Philipona, 2002). In such environments, even brief exposure to UVR during daylight hours may have significant lethal and sublethal impacts (Fu et al., 2022), selectively favouring behavioural regulation of UVR exposure. Moving forward, testing for UVR avoidance behaviour in other high elevation anuran species (Fu et al., 2022; Tang et al., 2022) might reveal behavioural modulation to be a common adaptation for mitigating harmful effects of UVR exposure. In parallel, it would be insightful to investigate potential mechanisms used by frogs to detect and monitor UV-A and/or UV-B. Knowledge in this area is limited, but vision may play a key role in controlling behavioural responses. Frogs are known to see in the UV-A spectrum, so it has been hypothesised that individuals may use UV-A as a signal to detect changes in UV-B (Han et al., 2007; Kats et al., 2012). Testing this hypothesis will be an important step towards better understanding the behavioural responses of anurans to UVR.

### Survival and growth

Our finding that UVR rearing environment can influence activity behaviour is particularly interesting when considered in the context of our survival and growth data. Throughout the study period, all 100 experimental animals survived, and the growth of sub-adult frogs in the two UVR treatments was statistically indistinguishable. Taken together, these results suggests that northern corroboree frogs may be using behaviour to regulate the effects of UVR exposure on critical fitness-determining traits. By maximising beneficial effects of UVR exposure (e.g. support for vitamin D_3_ synthesis and calcium metabolism) and minimising costs (e.g. damage to lipids, proteins cellular processes and DNA), individuals may be reducing the risk of mortality and optimising growth and development. Despite this implication, there are several limitations of our study that need to be considered. First, with the aim of preventing frogs from developing metabolic bone disease, we supplied them with a dietary vitamin D_3_ supplement throughout post-metamorphic development. As such, we cannot conclude that any beneficial effects of UVR exposure on calcium metabolism and growth were the outcome of UV-induced biosynthesis of vitamin D_3_ alone. Future work would benefit from assaying vitamin D_3_ levels in the blood serum of experimental frogs to ascertain the role of varying UVR exposure in vitamin D_3_ synthesis. In concert, it may be insightful to employ radiography to relate D_3_ levels to calcium metabolism and bone development. These approaches have been effectively used to study the influence of UVR exposure on growth and development in other frog species (Michaels et al., 2015; Tapley et al., 2015; Verschooren et al., 2011). Second, we have no data on whether UVR exposure stimulated physiological UVR defence responses that influenced the energy budget. Frogs are known to respond to UVR exposure by increasing UVR-screening compounds, increasing heat shock proteins, increasing anti-oxidant systems, and through DNA repair (Alton and Franklin, 2017; Fu et al., 2022; Hofer and Mokri, 2000). Investigating whether northern corroboree frogs invest in such physiological defence mechanisms, and whether minimising these investments has implications for growth, would allow deeper insight into the costs and benefits of UVR exposure and the optimal UVR range for captive breeding. In parallel, it would be valuable to quantify effects of UVR exposure on both embryos and larvae because UVR exposure can have strong impacts during these early life stages. Moreover, it would also be insightful to investigate whether UVR exposure during early life stages influences the performance and fitness of the individual post metamorphosis. Such carryover effects have been reported in several frog species (see Lundsgaard et al., 2023; Pahkala et al., 2001b).

To date, effects of UVR on the growth of anuran amphibians has been studied in various frog species, though the direction and magnitude of the reported effects has been highly variable. Past studies have reported that UVR exposure can have positive effects (Michaels et al., 2015; Pahkala et al., 2003; Tapley et al., 2015; Verschooren et al., 2011), negative effects (Belden et al., 2000; Belden and Blaustein, 2002a,b; Blaustein et al., 2003; Pahkala et al., 2001a, 2000; Smith et al., 2000) or no detectable influence on anuran growth (Antwis et al., 2014; Newton-Youens et al., 2022; Pahkala et al., 2003; Whatley et al., 2020). These differences may be linked to interspecific differences in a species natural habitat (e.g. tropical, temperate, alpine) and/or natural-activity patterns (e.g. diurnal, crepuscular, nocturnal or fossorial). Variation in experimental approaches employed may also influence experimental outcomes. For example, the direction and magnitude of effects might be influenced by interactions between irradiance levels, cumulative dose, exposure intervals and thermal environment (Hird et al., 2022; Lundsgaard et al., 2021). Furthermore, certain species might display UV hardening, whereby individuals acclimate to repeated UVR exposures, reducing impacts over time (Bancroft et al., 2008; Calfee et al., 2006). However, it is noteworthy that previous studies investigating anuran growth responses to UVR exposure have not simultaneously studied effects on activity behaviour. Therefore, from an ecological perspective, it is possible that effects may have been over- or under-stated, depending on whether experimental designs provided sufficient opportunity for individuals to self-regulate UVR exposure. With this in mind, we encourage future studies investigating the impact of UVR on amphibian growth to better explore interrelationships between behaviour and changes in body mass, body length, and skeletal morphology. In various frog species, there are direct links between body size and survival, fecundity, competitive ability and time spent active (Hoglund and Saterberg, 1989; Liao and Lu, 2009; Smith, 1987; Tejedo, 1992). Therefore, by using behaviour to optimise UVR exposure and growth, frogs might ensure developmental trajectories that significantly improve lifetime fitness. Moving forward, a combination of ecologically appropriate and mechanistic-focused behavioural and physiological multifactorial studies will be needed to build a holistic understanding of how UVR influences amphibian health and viability.

### Conservation implications

Our findings have important conservation implications. Northern corroboree frogs are listed as critically endangered by the IUCN, and captive breeding and reintroduction are priority recovery actions for this species (McFadden, 2013). Understanding the fitness impacts of UVR exposure regimes in artificial conditions is important for increasing the health and viability of captive animals. To this end, the data provided by this study will help inform the development of husbandry protocols and best-practice guidelines for conservation breeding. While we emphasise that our study cannot draw conclusions about what levels of UVR are harmful to northern corroboree frogs, the findings suggest that the consistent provision of UVR in the 0.1-0.75 range is sufficient to avoid issues that compromise growth (and development) and ensure high survival. Therefore, at present we recommend that northern corroboree frog CBPs supply post-metamorphic individuals with UVR in this range. Critically, however, given our findings suggest a capacity for behavioural regulation of UVR exposure, our recommendation comes with the caveat that rearing enclosures should contain refuge sites that allow frogs to regulate UVR exposure and intake.

More broadly, given that anuran amphibians are declining faster than any other vertebrate group, and that captive breeding is a standard recovery action for threatened species globally (Bradfield et al., 2023), ongoing research into the effects of UVR on amphibian fitness could potentially benefit conservation breeding programs for numerous threatened species. Based on current evidence, the provision of UVR has highly variable impacts on amphibians. While there is evidence for damaging effects of high UVR exposure, it must be highlighted that no or very low UVR can lead to various UVR-related health issues, including metabolic bone disease (Antwis and Browne, 2009). Given that amphibians vary greatly in their habitats, ecology and biology, much work is needed to gain a general understanding of UVR requirements for this vertebrate class. We stress that there is an urgent need for the acquisition of field data aimed at better understanding the range of UV-A and UV-B levels experienced by amphibians in nature, and experimental data aimed at understanding inter-relationships between UVR exposure, survival, growth, development, and avoidance behaviour. As more data become available across species, we will be better placed to design captive environments that expedite the implementation of conservation breeding programs to safeguard threatened species.

## MATERIALS AND METHODS

### Ethics statement

The procedures outlined below were performed following evaluation and approval by the Taronga Conservation Society Animal Ethics Committee (approval number 3c/12/22).

### Study species

The northern corroboree frog, *Pseudophryne pengilleyi,* is listed by the IUCN as critically endangered (IUCN SSC Amphibian Specialist Group, 2022) and in 2010, under the recommendation of the NSW Department of Climate Change, Energy, the Environment and Water (DCCEEW), a conservation breeding program for the species was established at Taronga Zoo (Sydney, Australia). While *P. pengilleyi* has been successfully bred in captivity for a number of years, there is lack of knowledge regarding the optimal UVR requirements for survival, growth and development. To date, rearing conditions utilising UV-B fluorescent lighting have produced healthy offspring at between 0.2-0.3 W/m^2^ (measured at ground level) (McFadden et al., 2013). However, frogs exposed to UVI of over 1.4 have demonstrated an abnormal increase in black pigmentation and skin damage (McFadden, personal observation).

### Experimental animals

A total of 100 captive bred *P. pengilleyi* (Fig. 1A) were reared in captivity at Taronga Zoo, Sydney, NSW, Australia. Frogs within the captive assurance colony are separated according to the location of the wild source populations of founding individuals, with offspring used in the present study generated from breeding stock from the northern Brindabella distribution of the species. Frogs were reared from 12 clutches (each produced by separate male-female pairs). The experimental animals were produced as eggs in March 2022 and communally reared as tadpoles in two large tanks, with clutches split between tanks. Tadpoles were fed on a diet of natural silt, frozen endive and powdered Fluval vegetarian fish flake. Food was provided *ad libitum* to ensure that tadpoles were fed to satiation. Tadpoles were maintained in reverse osmosis-filtered water with the addition of diluted trace elements (0.03 ml/l of Seachem Fresh Trace, USA). The bottom-dwelling tadpoles had access to very low levels of UVR (<0.1 UVI) (Reptisun T5HO 5.0 UVB bulb; Pet Pacific, Australia), with tubes suspended 35 cm above water level and water depth of 20 cm. New globes were installed 1 week prior to tadpole rearing to allow UVR levels to stabilise. All experimental animals completed metamorphosis (point of full tails absorption) between November and December 2022. Metamorphs from each tadpole rearing tank were randomly allocated to the UVR treatment containers at approximately 15-40 days post metamorphosis. Individual metamorphs were identified by their unique dorsal and ventral patterns so that the growth of individuals could be tracked. During the study, all post metamorphic animals remained in the subadult life stage.

### Experimental design

To investigate the influence of UVR levels on activity, survival, and growth individual *P. pengilleyi* were reared under one of two UVR treatments: a higher UVR treatment (UVI mean±s.e.m=0.75±0.032) and a lower UVR treatment (UVI mean±s.e.m=0.20±0.017). These UVR levels were selected because they represented the two alternate ends of the Ferguson Zone (Zone 1, 0-0.7 UVI) that may be applicable to the natural behavioural activity of this species (Baines et al., 2016; Ferguson et al., 2010). Specifically, while *P. pengilleyi* is primarily nocturnal, the species will display crepuscular activity, as well as diurnal activity under shaded (low light) conditions. UVI levels were measured weekly from underneath the experimental container lid using a Solarmeter 6.5 (USA) while UV-B and UV-A levels were measured with an Alliance Technologies SpectroSense 2 (+) radiometer to ensure consistency in UVR output throughout the study period. The differences in UVI levels between treatment containers was significant (ANOVA: *F_1,17_*=205.48, *P*<0.001). The treatments also showed significant differences in UV-B levels (W/m^2^) (higher UVR treatment mean±s.e.m=0.277±0.019; lower UVR treatment mean±s.e.m=0.093±0.003; ANOVA: *F_1,16_*=91.70, *P*<0.001), and UV-A levels (W/m^2^) (higher UVR treatment mean±s.e.m=1.118±0.083; lower UVR treatment mean±s.e.m=0.380±0.009; ANOVA: *F_1,17_*=77.29, *P*<0.001). Each treatment had a sample size of 50 frogs (*n*=100 experimental individuals in total). For each treatment, animals were housed in groups of five in plastic experimental containers (30 cm×19 cm and 20 cm high), that were secured with a ventilated flyscreen mesh lid which allowed for UV light penetration (Fig. 1B). The 20 experimental containers (ten per treatment) were positioned across four shelves in a room; two shelves housed the lower UVR treatment containers (five containers per shelf) and the other two shelves housed the higher UVR treatment containers (five containers per shelf). To avoid any potential room positioning effects, each experimental container was moved two positions to the right every 4 weeks, so that each experimental container rotated through all possible positions within the two respective treatment shelves throughout the experimental period.

On day 1 of the experiment, frogs were weighed to the nearest 0.01 g using a digital scale, and body length measured to the nearest 0.01 mm using a set of analogue vernier callipers. Frogs were then randomly assigned to treatment containers (lower UVR or higher UVR). The experimental room was artificially illuminated, controlled via a light-sensitive switch to replicate natural photoperiod. Skylights and a window within the facility permitted a natural dawn and dusk. In addition to overhead room lighting, UV-B lights (Reptisun T5HO 5.0 UVB bulb; Pet Pacific, Australia) were suspended approximately 25 cm above each container. New globes were installed 1 week before the experiment started to allow UVR levels to stabilise. Frogs in the higher UVR group had a reflector positioned above the tube to achieve the higher level of UVI delivered. These lights were also controlled by the light-sensitive switch, providing approximately 12 h per day of UV light (between 7 am and 7 pm). Incident visible light levels (lux) were measured with a digital light meter (Jaycar, QM1587). Of note, there was a significant difference in visible light (lux) levels between the two treatments (higher UVR treatment mean±s.e.m=130.777±9.217; lower UVR treatment mean±s.e.m=64.333±1.178; ANOVA: *F_1,16_*=51.13, *P*<0.001). Ambient temperature in the room cycled from 20°C during the day hours to 17°C at night and was constant across all treatment containers. There was no significant difference in temperature between the experimental treatment containers, either during the night time (higher UVR treatment mean±s.e.m=14.96±0.047; lower UVR treatment mean±s.e.m=14.98±0.045; ANOVA: *F_1,17_*=0.115, *P*=0.74) or the daytime (higher UVR treatment mean±s.e.m=17.46±0.046; lower UVR treatment mean±s.e.m=17.48±0.046; ANOVA: *F_1,17_*=0.11, *P*=0.73). These conditions were in accordance with corroboree frog husbandry procedures developed by Taronga Zoo. The experimental period lasted 114 days, starting on 22 December 2022 and finishing on 16 April 2023.

### Husbandry and nutrition

During the experimental period, frogs were fed three times a week with 2-4-day-old crickets (*Acheta domestica*). This feeding regime ensured that food was available *ad libitum*. In addition, to ensure healthy bone growth, all individuals received calcium and multivitamin supplementation (containing vitamin D_3_) by lightly dusting the crickets (Repashy Calcium Plus, USA). During the experiment, containers housing frogs were lined with a substrate of aquarium gravel 2 cm deep, with half of the floor space covered in a 5 cm layer of rehydrated sphagnum moss (Brunnings, Australia). All enclosures were sprayed with reverse osmosis-filtered water twice a week, on Tuesdays and Saturdays, to flush through any uneaten food items and excrement.

### Quantifying activity

For a period of 112 consecutive days (24 December 2022-16 April 2023) the activity of frogs was scored. Of note, the activity assay started 2 days after the experiment started to allow frogs to acclimate to the experimental containers. Once a day, between 07:15 h and 11:45 h (mean±s.e.m=8.49±0.11, min=06:30 h, max=11:45 h) all experimental containers were observed for a 3-min period and the number of frogs active on the surface of the substrate under the UV light were counted. Observations were made by five Taronga Zoo staff from the herpetofauna division, with observers randomly alternating between days. To avoid disturbance to frogs, all counts took place during the first instance that the experimental room was accessed for the day. The order that containers were observed was randomised, alternating between containers from each treatment group.

### Quantifying survival and growth

To quantify frog survivorship, containers were carefully checked every 2 weeks and frogs were scored as alive or dead. To quantify growth, each individual was weighed every 2 weeks over the experimental period, equating to eight repeat samples (weeks 2, 4, 6, 8, 10, 12, 14 and 16). At each repeat sample, frogs were removed from their experimental containers and weighed to the nearest 0.01 g using a digital scale. The SVL [body length measured from the tip of the nose (snout) to the bottom of the anus (vent) in mm] was measured for each individual using a set of analogue vernier callipers.

### Statistical analyses

On day 1 of the experiment, differences in the body mass and body length of frogs in each treatment group were compared using one-way ANOVAs. The effect of UVR treatment level (higher UVR and lower UVR) and rearing time (experimental day) on frog activity level (proportion of frogs active on the surface per experimental container) was tested using a linear mixed effects (LME) model fitted with restricted maximum likelihood (REML). UVR treatment (higher or lower) was entered into the model as fixed categorical effect, while the experimental day (1-112) was included as a continuous variable, and the experimental container number (1-20) was included as a random effect (to account for repeated measures). The effect of UVR treatment (higher UVR or lower UVR) on frog body length (SVL, mm) and body mass (g) at experimental weeks 2, 4, 6, 8, 10, 12, 14 and 16 were tested using two separate LME models fitted with REML. In each model, UVR treatment (higher UVR or lower UVR) and experimental week were entered as fixed categorical effects, while frog ID was included as a random effect (to account for repeated measures). Prior to analysis, body weight and body length data were log transformed, and activity data (proportion of frogs active on the surface) was ARCSINSQRT transformed [using the formula sin^−1^(√x)] to improve normality and stabilise variances. All statistical analyses were performed using JMP 16.0 software package (SAS Institute Inc. NC, USA).
